# Fireless Locomotives

**Published:** 1881-07

**Authors:** 


### FIRELESS LOCOMOTIVES.

Improvements in detail have been made by M. Leon Francq, who lately read a paper on the subject before the French Association for the Advancement of Science, from which we glean the following particulars: The locomotive is provided with a tank containing water at a sufficiently high temperature (203° cent., equal to 397° Fahr.) to produce the necessary quantity of steam for the journey. The water is heated at the starting point by means of a jet of steam at high pressure produced by a stationary boller. As the boiling point increases with the pressure, it follows that, in a closed vessel, the greater the heat the higher the pressure attained. If the heating be effected by a jet of steam, as in the present case, the steam fills the space above the surface of the water, at the same time increasing the pressure. To apply this principle it is sufficient that the tank stand a pressure of from two to fifteen atmospheres (30 to 225 pounds per square inch). The steam from the stationary boiler fills three parts of the receiver and agitates the water sufficiently to distribute the heat uniformly. When an equilibrium of pressure between the boiler and the receiver is attained the cocks are turned off. The locomotive is then in running order, ebullition taking place directly communication is opened between the tank and the cylinders.

In practice the initial temperature may attain 200° Cent. (392° Fahr.), which corresponds to fifteen atmospheres or 225 pounds per square inch. The final pressure must be sufficient to take the train up the steepest gradient to be encountered. The tank or receiver is made of steel plates, and may contain over 1,800 liters (396 gallons). After leaving the receiver the steam passes into an intermediate chamber, which allows the steam to expand so as to enter the cylinders at a uniform pressure, independent of that in the tank or receiver. The exhaust steam is not utilized as in the ordinary locomotive, because there is no fire to urge, but escapes into an air condenser which is a closed cylindrical vessel traversed by more than 600 tubes open at both ends. The water of condensation passes into a tank, whence it is afterward withdrawn as feed water. The diameter of the cylinders is 23 centimetres (9 inches), and the length of stroke 25 centimeters (9 7-8 inches), the working parts not differing from those of ordinary engines. The weight of the engine running light is 6¾ tons; and the tractive power is from 343 kilos (6¾ cwt.) to 1,031 kilos (1 ton), according to the pressure. In the event of an unusual resistance being encountered on the road it is sufficient to act, by a rod and lever, on the intermediate or equalizing chamber, so as to give a temporary increase of pressure on the pistons. At a speed of 12 kilometers (7½ miles) an hour, the wheels which are 75 centimeters (1 foot 5½ inches) in diameter, make 86 revolutions in a minute. With a stationary boiler of about 50 square meters (538 square feet) of heating surface, a working pressure may be maintained in the locomotive seventeen or eighteen minutes. The consumption of fuel is found by experiment to be less for a given duty than is the case of ordinary locomotives. In a line of 10 kilometers (over 6

miles), the working expenses, including repairs and depreciation of stock, amounted to 45½ centimes per kilometer—say 7d. a mile run.—*Scientific American.*

